# 178 Pace and Pitch: Predictive Factors for Seed Funding and Development – CORRIGENDUM

**DOI:** 10.1017/cts.2024.634

**Published:** 2024-10-22

**Authors:** Alyson Eggleston, Camelia Kantor

The above abstract published with an error in the author list. The correct list is shown below.

Alyson Eggleston and Camelia Kantor
